# Comparative study on the performance of solar still equipped with local clay as an energy storage material

**DOI:** 10.1007/s11356-022-21095-z

**Published:** 2022-06-01

**Authors:** Ahmed H. Mohammed, Mohamed Attalla, Ahmed N. Shmroukh

**Affiliations:** Department of Mechanical Engineering, South Valley University, Qena, 83521 Egypt

**Keywords:** Solar still, Energy storage, Local clay, Phase change materials, Desalination

## Abstract

The paucity of freshwater is very dangerous in the coming years. Many coastal countries suffer from a scarcity of freshwater. Solar desalination is the cheapest way to produce freshwater from any type of non-drinkable water (brackish water and seawater). In this work, single-slope single-basin solar still for seawater desalination was examined under Upper Egyptian weather conditions of Qena City (latitude 26.16°, longitude 32.71°). The main goal of the work is to compare the performance of conventional solar still, solar still supported with PCM, and solar still supported with local clay material to augment the solar still yield during both daytime and nighttime periods of operation. The results demonstrated that the total production of desalinated water from the simple conventional solar still, the solar still with PCM, and the solar still with local clay reached about 3885, 4704, and 5388.6 ml/m^2^, respectively. Moreover, compared to the conventional solar still, the yield was increased by about 21% when using the PCM, and about 38.7% when using the local clay material. Furthermore, it can be observed that the daytime productivity in the case of solar still supported with local clay was higher than that for the solar still supported with PCM, while the nighttime productivity was higher in the case of solar still supported with PCM compared with solar still supported with local clay. Moreover, the average daily efficiency of conventional solar still, solar still with PCM, and solar still with local clay reached about 34, 41.2, and 47%, respectively. Therefore, it is recommended to use the solar still with local clay for seawater desalination in such arid and hot climate of Qena City.

## Introduction

Today, freshwater shortage is increasing continuously, because of industrial prosperity, increase of agricultural reclamation, and population increase (Nisan and Benzarti 2008). About 70% of the earth is covered by water, while sea and ocean water represent 97% of the earth’s water. Furthermore, freshwater forms only 3% of the world’s water represented in glaciers, underground, rivers, and lakes (Schmitt 1995; Elfasakhany 2016). Desalination processes help to solve this problem by using various types of non-renewable (Tian et al. 2005; Kavvadias and Khamis 2014) or renewable energy (Xiao et al. 2013) technologies. Numerous researchers agreed that solar desalination is the cheapest way to produce freshwater from any type of non-drinkable water (brackish and seawater) (Mohammed et al. 2021). Solar still is a simple installation device used to desalinate seawater using solar energy. However, the yield of the conventional solar still (CSS) is low and it is related to the daytime solar radiation; this means that the daily yield of solar still is limited to the brightness of the sun (Hermosillo et al. 2012; Zhang et al. 2013). Several studies have investigated various ways to overcome the proposed problem (Shukla and Sorayan 2005; Jathar et al. 2021). Using stored energy is one of the best techniques used to recover the heat from any thermal applications. Energy can be stored in two main forms of changing the phase transformation of material (as in the case of latent heat thermal energy storage—LHTES) or changing the internal energy of a material (as in the case of sensible heat thermal energy storage—SHTES) (Fath 1998; Elfasakhany 2016; Dhivagar et al. 2020a, b). These techniques can be utilized to enhance the performance of thermal systems. Phase change materials (PCM) such as inorganic, organic, and eutectic substances can be used as latent heat storage material. Gravels, mild steel scraps, eggs shells, egg shell powder, gravel sand, El Oued sand grains, dye, pebbles, iron chips, and sand can be used as sensible heat storage (Murugavel et al. 2008, 2010; Samuel et al. 2016; Dhivagar et al. 2020a, b; Balachandran et al. 2021; Dhivagar et al. 2021; Dubey and Mishra 2021; Attia et al. 2022; Thakur and Sathyamurthy 2022). However, PCM had a bigger impact on the solar desalination (El-Sebaii et al. 2009a). Many researchers were headed for enhancing the effectiveness of solar still through various techniques and tried to increase their yield. Kabeel et al. (2018b) enhanced the solar still efficiency by utilizing jute cloth with sand as sensible heat storage material. Identically, Dumka et al. (2019) used cotton bags filled with sand to enhance the solar still efficiency. Also, Kabeel et al. (2019a) used composite backed material of black gravel with phase change material to enhance the solar still performance. Moreover, Asbik et al. (2021) investigated the performance of solar still with sand and paraffin wax to enhance the thermal efficiency. Other studies investigated different material inside solar still to enhance the performance of solar still such as sand and paraffin wax, aluminum balls, rehash cooked oil, and nano-ferric oxide (Attia et al. 2020; Balachandran et al. 2020a, b; Prasad et al. 2021). Vigneswaran et al. (2019) investigated the performance of the solar still with a single PCM and multiple PCMs to enhance the temperature difference between water and glazing surface to increase the productivity of the solar still. The maximum yield of their proposed still reached about 4.40 L/m^2^/day. Mohamed et al. (2019) improved the solar still total yield using basalt stone as a sensible heat storage material and improve the yield over conventional simple solar still by about 33.7%, while Modi and Modi (2019) studied the solar still performance with different water depths using heat storage and regenerative medium. Their results demonstrated that the total yield could be improved by 18% and 24% for 1-cm and 2-cm water depth, respectively. Akash et al. (1998) utilized different thermal storage materials such as dark black ink, black rubber matt, and black dyes inside the solar still. The maximum productivity obtained in case of using black rubber mate improved by 38% increment in desalinated water production. Attia et al. (2021a, b) used phosphate bed as energy storage material to enhance the performance of CSS, and Attia et al. (2021a, b) studied the performance of solar still with cotton bags filled with phosphate inside basin area. Sakthivel and Shanmugasundaram (2008) studied the solar still performance by using thermal storage material of black granite gravel with different depth of the gravel. It is found that the desalinate output was enhanced by about 17%. Kabeel et al. (2019b) used cement-coated red bricks as an energy storage material inside solar still for increasing its freshwater production. Cement-coated red bricks were kept inside the basin area. They found that there was an improvement of about 34% for the water temperature in the case of modified solar still at 20 kg water mass, which acts as water evaporation driving force inside the closed chamber. Furthermore, the yield was improved by 45% as compared to the CSS (Kabeel et al. 2019b). Another study used graphite to investigate its effect on the performance of the solar still (Kabeel et al. 2018a). They found that the daily yield was reached about 7730 ml/m^2^, and 4410 ml/m^2^ for solar still with graphite, and conventional solar still, respectively (Kabeel et al. 2018a). One of the methods of energy utilization improving is the storing of energy during sunshine times of higher solar radiation for later use of necessary needs. To meet energy demand, efficient storage technologies are essential to store the required energy for 24-h cycles. Energy storage helps match the energy demand peaks and power generation for night periods (Ravi Gugulothu et al. 2015). Jani and Modi (2019) evaluated solar still of double slope and single-basin type equipped with square and circular hollow fins. They found that the 10-mm basin water depth produced a maximum output yield of 0.9672 and 1.4917 kg/m^2^ day when using the square-finned and circular-finned solar still, respectively (Jani and Modi 2019). El Hadi Attia et al. (2021) studied the performance of hemispherical solar still with iron fins with different length and spacing in absorber plate, to increase the absorption area and to enhance the basin water temperature, while El Hadi Attia et al. (2021) proved that a specified amount of heat to the saline water with constant rate is more effective than the same amount of heat in random mode.

According to that state of the art, it is found that some researchers used latent and sensible heat material for increasing the solar stills’ productivity, while there were no studies in the literature focused on using the Egyptian local clay in solar still.

The novelty of the present research work is to improve the solar still productivity by using a type of red Egyptian local clay conjoined with activated carbon particles on the absorber area of the solar still. Such novel combined material should enhance the sensible heat energy storage during the daytime and then takes advantage of it during the nighttime. To achieve this aim, experiments were conducted using three types of solar stills. The first one is conventional solar still (CSS) without any modification, while the second one contains 4 kg of PCM (SSPCM), and the third one contains 4 kg of local clay (SSLC).

## Experimental setup

### The proposed test rigs

**Fig. 1.**
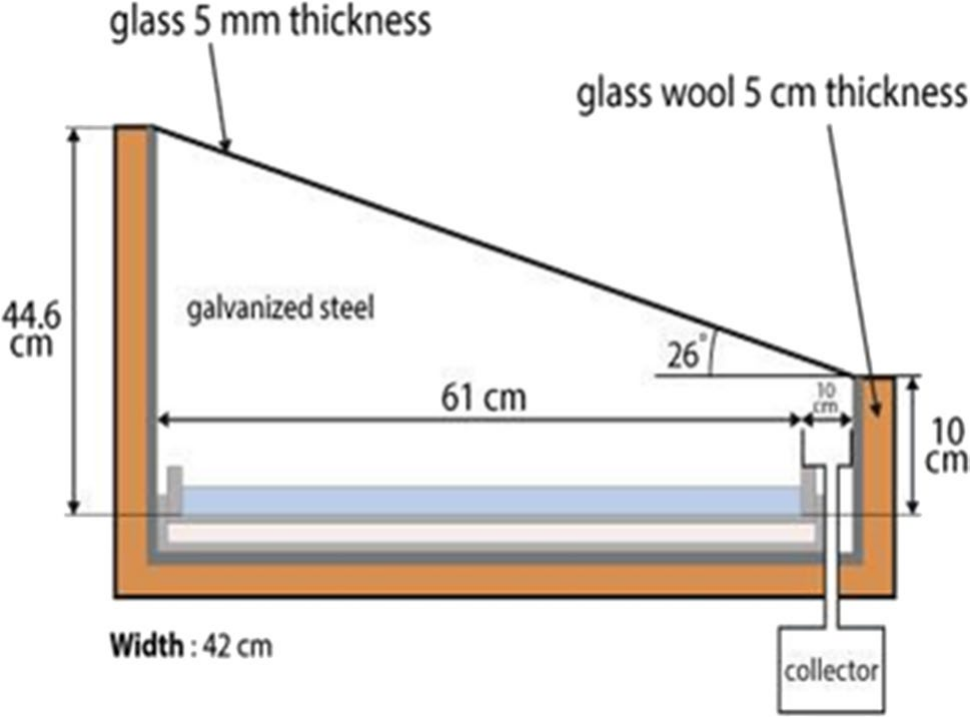
Proposed solar still dimensions

**Fig. 2.**
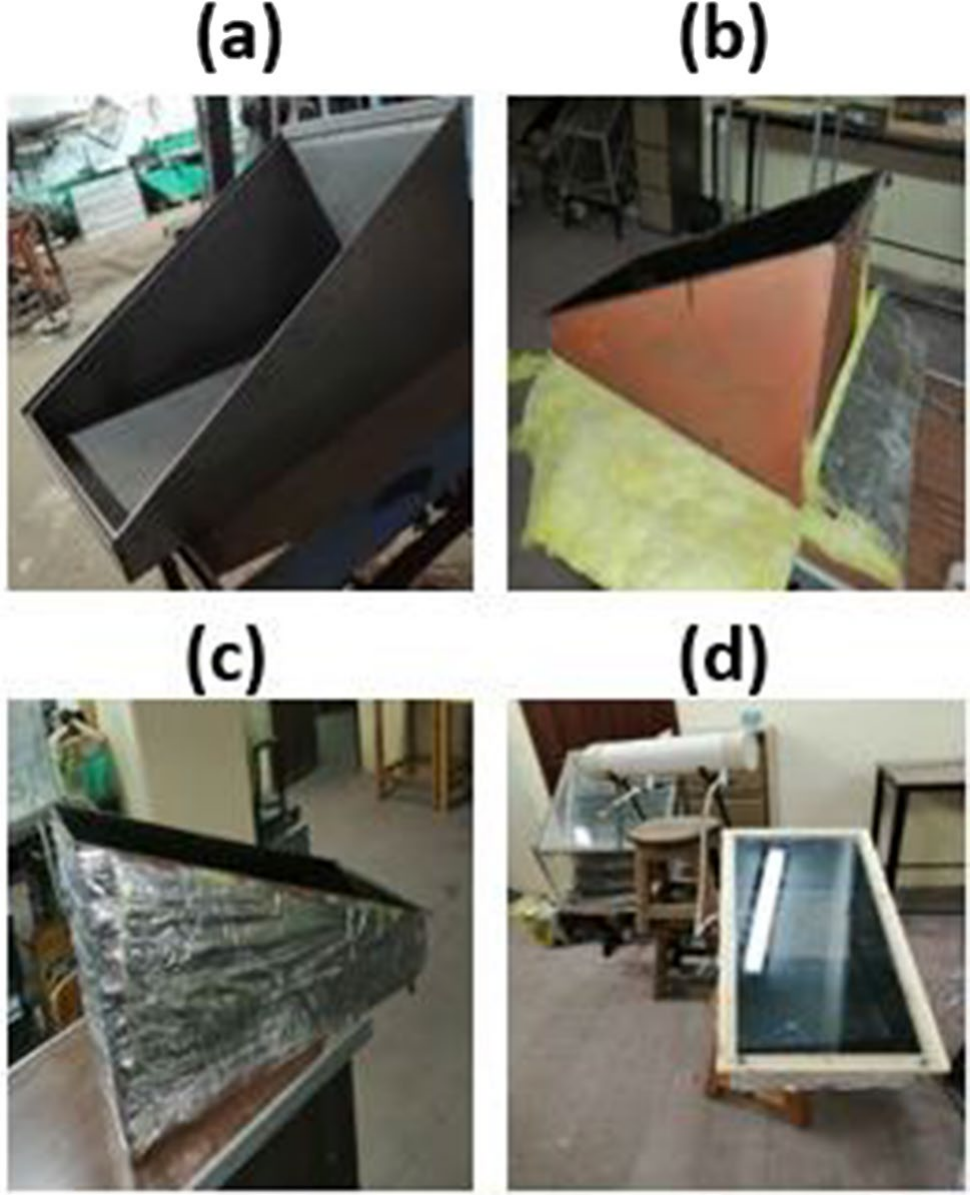
Solar still manufacturing stages. **a** Iron sheet assembly, **b** basin walls painting, **c** glass wool insulation, and **d** glass cover fixation

Single-slope solar still for seawater desalination was examined under Upper Egyptian weather conditions of Qena City (latitude 26.16°, longitude 32.71°). The construction of the still was made in the workshop of the Mechanical Engineering Department, Qena Faculty of Engineering, South Valley University. The basin-type solar still was constructed with a galvanized iron sheet with 1-mm thickness. The bottom and the sides of the iron box were insulated by glass wool insulation; the thickness of glass wool insulation was 5 cm for all sides. The dimensions of the solar still iron box are shown in Fig. 1. Furthermore, the stages of the solar still manufacturing are shown in Fig. 2. The thickness of the glass sheet is 5 mm which is used as a transparent cover. A channel was fixed under the glass cover lower part for collecting the condensate. A small plastic pipe is fixed at the tip of the collecting channel to drain the freshwater into the external vessel. The proposed solar still glass cover was mounted at an optimum angle of 26° to ensure that the condensed water will run down to the channel of the freshwater collector (Nasr and Shmroukh 2020). The absorbing plate is also made of galvanized iron sheet with a 1-mm thickness which has dimensions of 61 cm × 42 cm, and the solar still effective area is approximately 0.25 m^2^. The solar still absorbing plate has an area of 0.25 m^2^ and it was painted with high absorptive black paint. Afterward, three holes were drilled to the solar still, one of them for seawater inlet, the second for condensed water outlet, and the third for discharging of the remaining brine. After the construction of the solar still, it was painted with black paint. After the painting process was finished, the insulation material was fixed on all sides of the solar still body. The channels were stuck to the glass cover and then the glass cover was settled onto the construction. Seawater depth was adjusted to 0.5 cm and using the storage tank to compensate for the shortage in water depth inside the solar still basin (Mohamed and Shmroukh 2020). Thermocouples were placed in different places of the still before fixing the glass cover, to record different temperatures, such as inside glass cover, basin water, and inside moist air temperature. A data logger was used to record the temperature values. Silicon rubber band was used to ensure that no air will escape through small spaces between the cover and still.

**Fig. 3.**
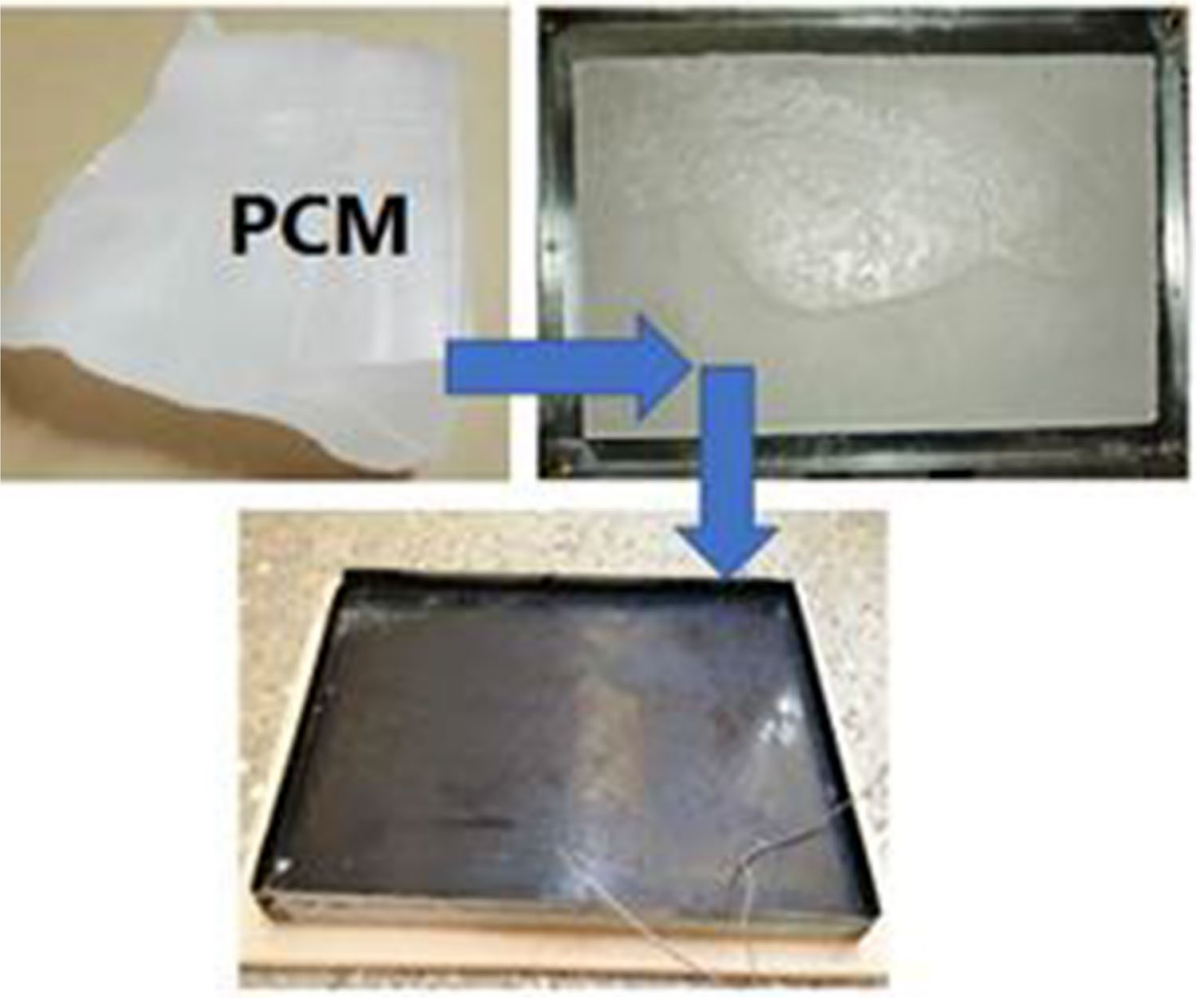
Stages of PCM container fabrication

Phase change material was placed inside the container by converting it to a liquid phase using a heat source and then poured it inside the container, then closed the external cover of the container and put the silicon as a seal. Figure 3 shows the stages of the container fabrication. After that, the container is placed inside the proposed solar still. More detailed information about the designed system can be found in the previous study of the authors (Mohammed et al. 2021).

**Fig. 4.**
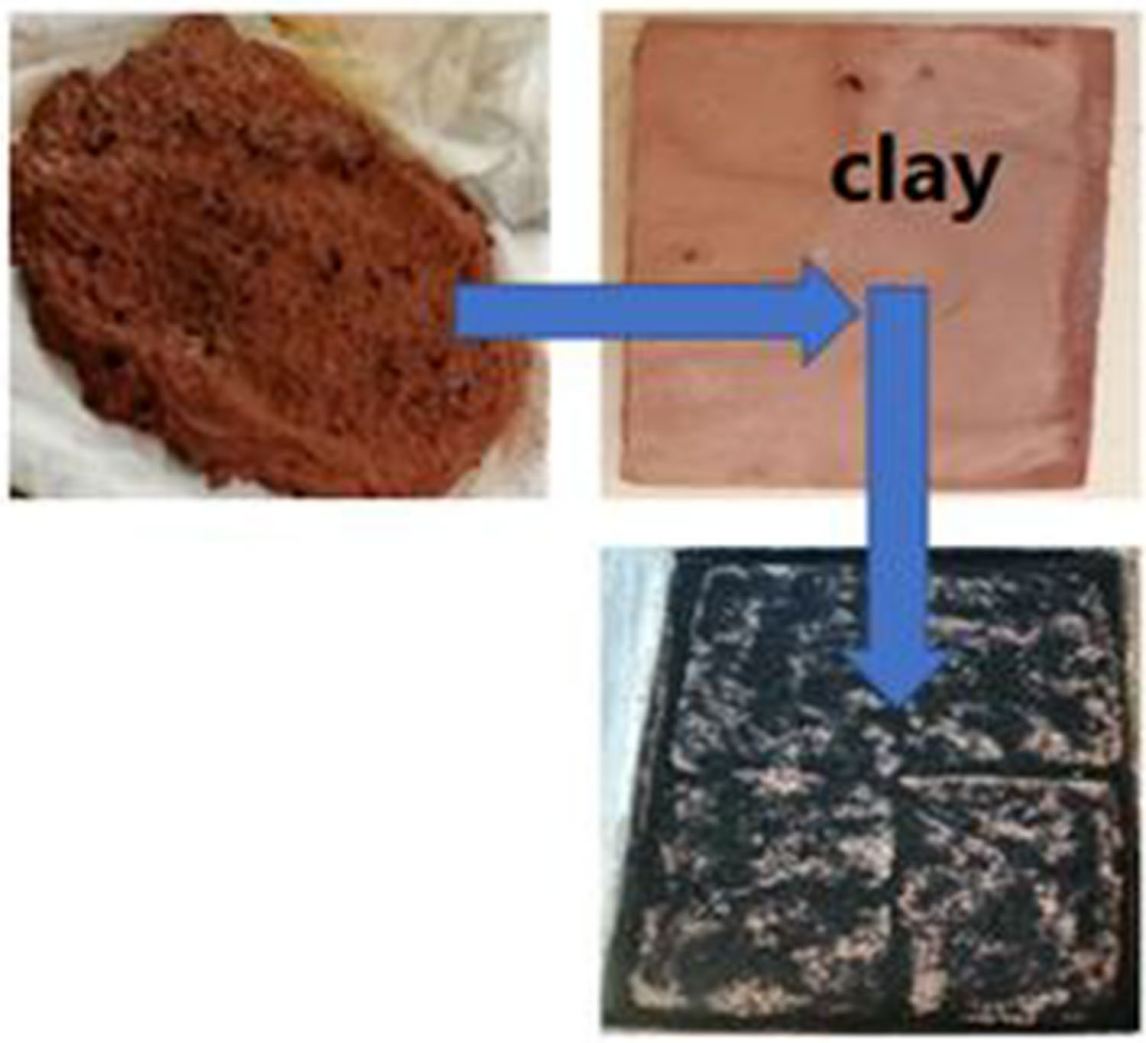
Stages of the local clay material fabrication

**Fig. 5.**
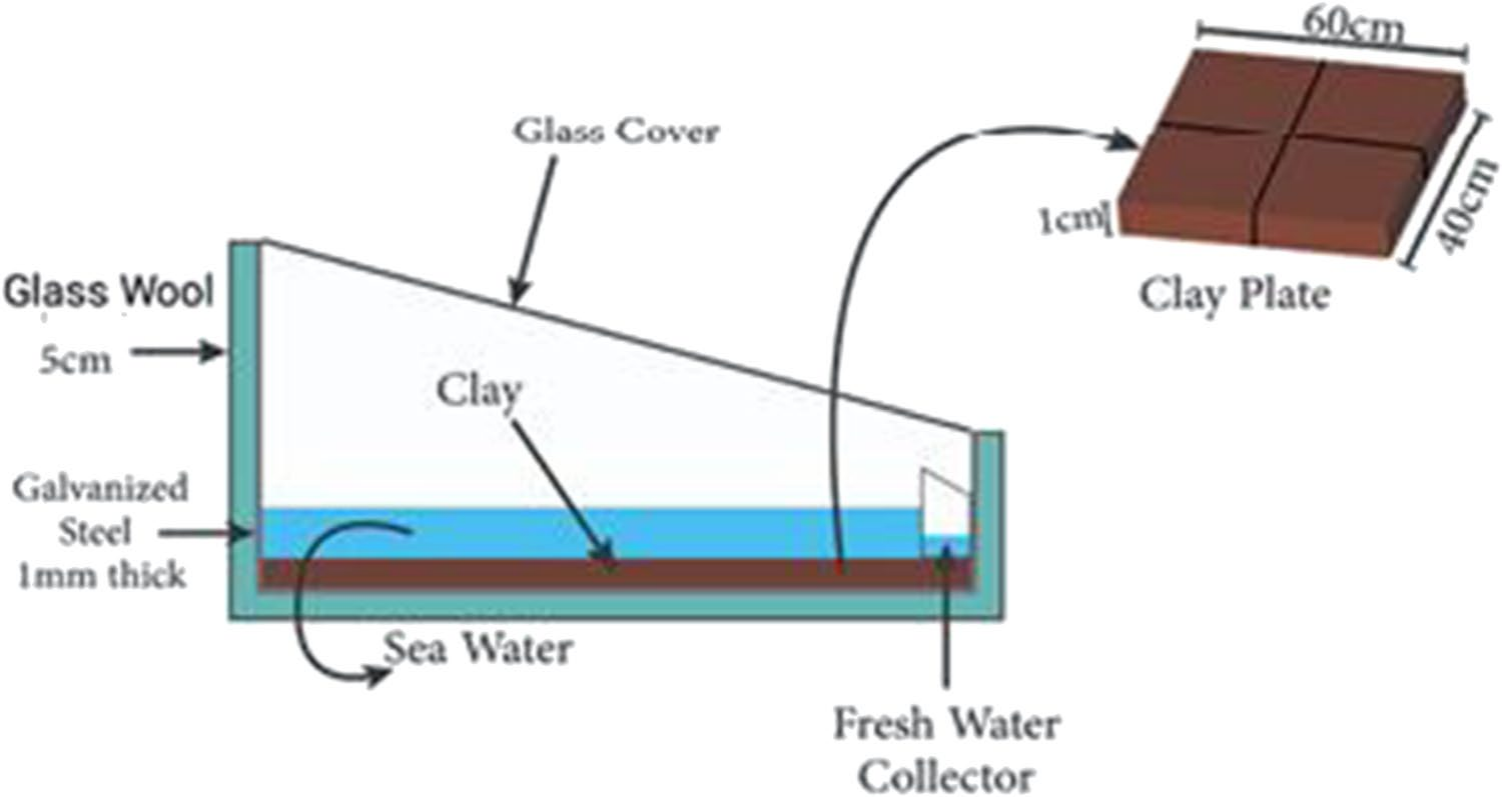
Schematic layout of the SSLC

Local clay was made from Aswan clay balls in the Faculty of Specific Education laboratories, South Valley University. Figure 4 shows the stages of the local clay fabrication along with adding activated carbon to increase solar radiation absorptivity (Baioumy and Ismael 2014). Moreover, Fig. 5 shows the solar still with local clay material.

The proposed Egyptian local clay density is 1.692 g/m^3^, porosity is 22.9%, and specific heat is about 1.343 kJ/kg °C. Furthermore, using X-ray fluorescence analysis, the elemental composition of the proposed Egyptian local clay consists of several elements such as 15.79 Wt% of iron (Fe_2_O_3_), 8.5 Wt% of aluminum (Al_2_O_3_), 6.5 Wt% of magnesium (MgO), 0.35 Wt% of manganese (Mn_2_O_3_), and 0.89 Wt% of titanium (TiO_2_), respectively. Table 1 represents the complete chemical composition of the used local clay.

**Table 1:** Local clay composition

Composition of sample	Concentration (Wt %)
MgO	6.5
Al_2_O_3_	8.5
Fe_2_O_3_	15.79
Mn_2_O_3_	0.35
TiO_2_	0.89
CaO	11.2
K_2_O	0.33
SiO_2_	45.1
Cl^-^	0.2
SO_3_	9.8
P_2_O_5_	0.87

### Desalination unit

**Fig. 6.**
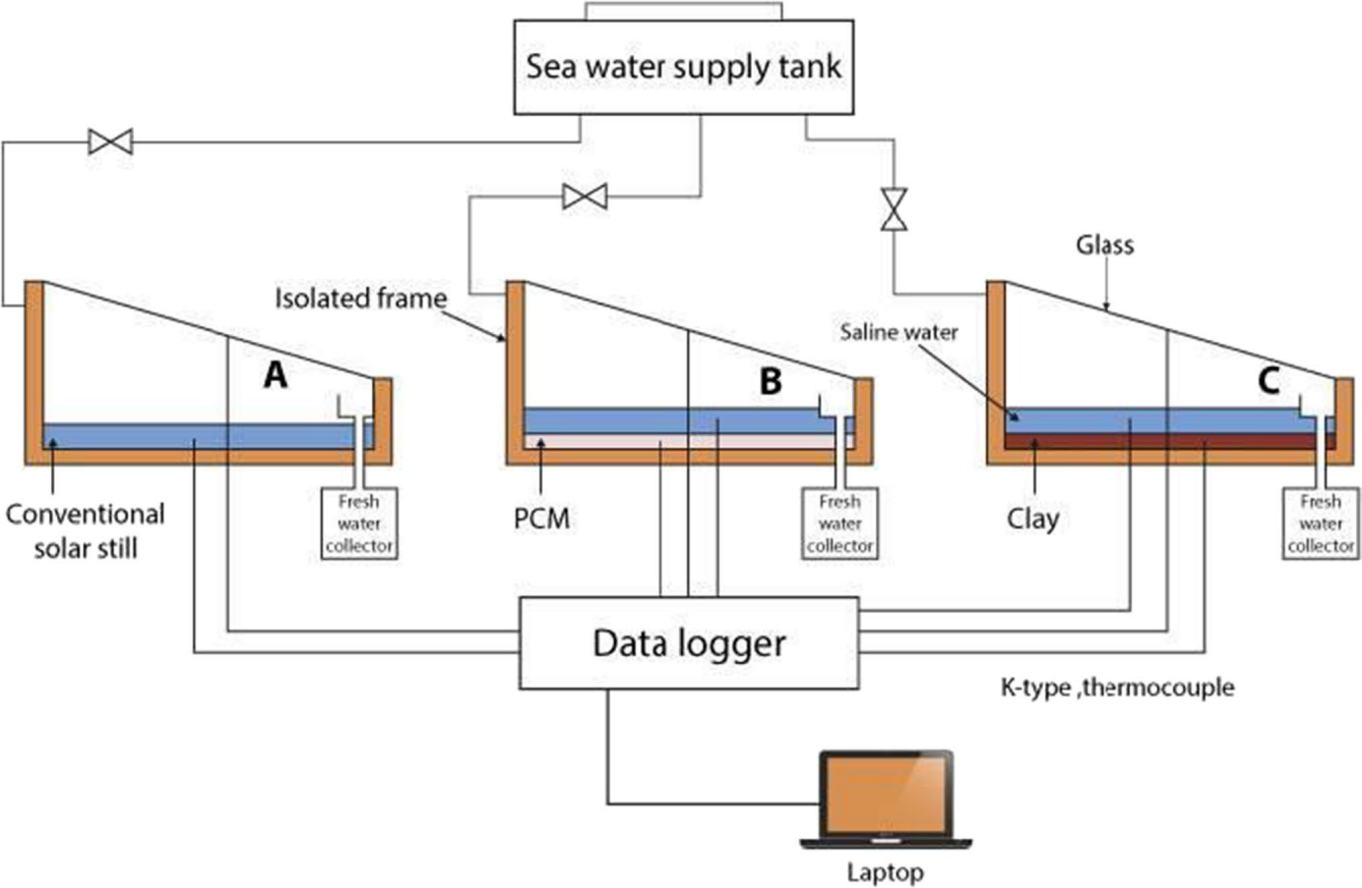
Schematic layout of the experimental setup

**Fig. 7.**
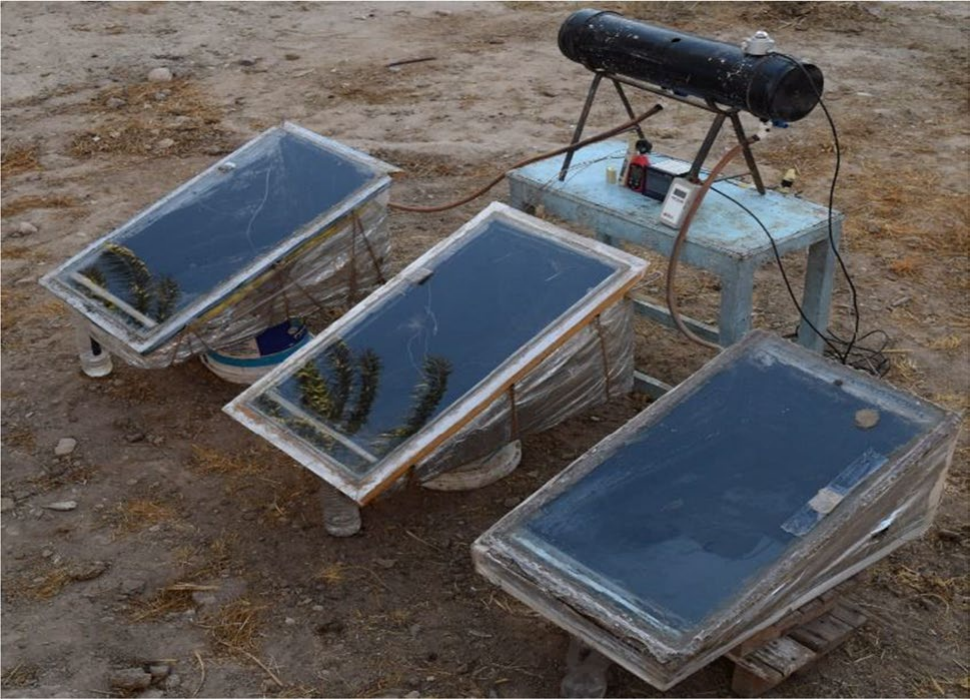
Photographic view of the experimental setup

In the present work, three solar stills were used, to examine different approaches. Figure 6 shows a schematic diagram of the three solar stills. The first distiller is a conventional solar still (CSS), the second one is a modified solar still using 4 kg of PCM (SSPCM), and the third one is a modified solar still using 4 kg of the proposed Egyptian local clay (SSLC). Figure 7 shows a photographic view of the proposed solar stills. The purpose of the study is to investigate the energy storage behavior of both the clay and PCM to improve the solar still productivity during both daytime and nighttime operation. The performance of the three solar stills was observed during June 2020 from 7 a.m. to 8 p.m.

### Instrumentation

The quantity of the desalinated water was measured every 1 h during all experiments. Temperatures of the inner surface of the glass cover, ambient air, clay, PCM, and basin seawater and the wind speed, solar radiation intensity, humidity, and the desalinated water production were measured each hour. Table 1 shows the specifications of the measuring instruments. The procedure of Shmroukh et al. (2019) for the calculation of both uncertainty and error that occurred in the measuring instruments and in the experimental results was used and clarified in this subsection. Therefore, the following equation is used for calculating the mean of the quantity measured in the experiments (Omara et al. 2011):

1 $$\dot{\mathrm{x}}=\frac{1}{N}{\sum}_{i=1}^N xi$$

Furthermore, the random error is estimated using statistical analysis and the standard deviation equation is followed (Shmroukh et al. 2021):

2 $${\sigma}_x=\sqrt{\frac{1}{N-1}\sum\nolimits_{i=1}^N{\left({x}_i-\overline{x}\right)}^{2\kern0.5em }}$$

Furthermore, the determined values’ error can be obtained by applying the uncertainty propagation as follows (Shmroukh 2019) :

3 $$\delta R=\sqrt{{\left[\frac{\partial R}{\partial {x}_1}\delta {x}_1\right]}^2+{\left[\frac{\partial R}{\partial {x}_2}\delta {x}_2\right]}^2+\dots .+{\left[\frac{\partial R}{\partial {x}_N}\delta {x}_N\right]}^2}$$

The obtained values from the uncertainty analysis are accompanied by the specifications of the used instruments and are illustrated in Table 2.

**Table 2:** Measuring instruments specifications

Instrument	Manufacturer/model	Range and error
Wind speed	AnemometerUT363	0–108 km/h, ± 5%
Thermocouple	K-type	− 50 °C to 150 °C, ± 0.1%
Digital pyranometer	Kipp & Zonen CM4	0–4000 W/m^2^, ± 0.93%
Humidity	Hygrometer	0–100%, ± 5%
Water clarity	TB400	± 5%
Combined meter (pH /TDS)	Mi 806	pH range: 0 to 14 pH TDS range: 0 to 50000 ppm, ± 2%
Beaker	PLASTI BRAND Germany	0–200 ml, ± 0.12%

## Results and discussion

**Fig. 8.**
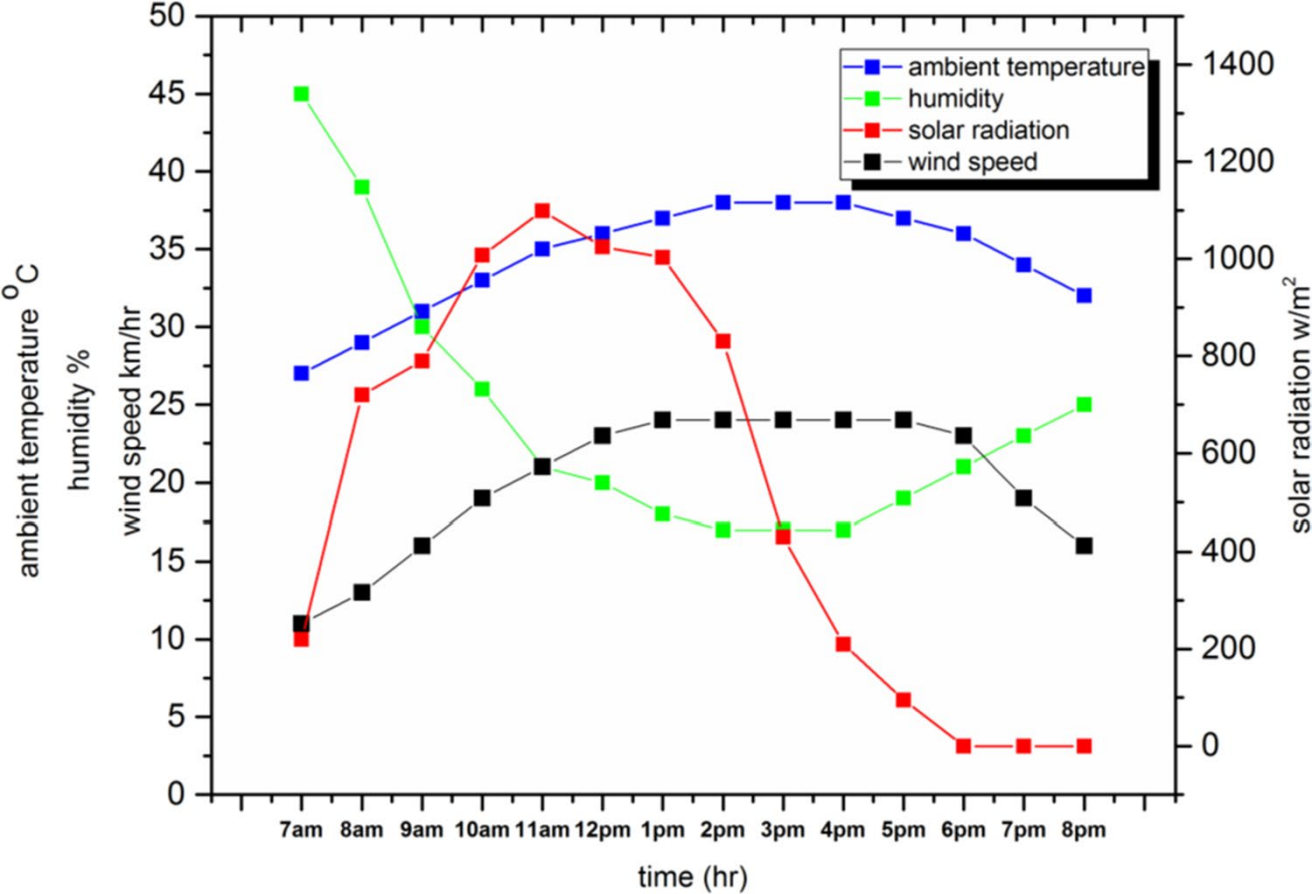
Environmental conditions during the experiment

The experiments were conducted using three types of solar stills, the first one is conventional solar still (CSS) without any modifications, the second one contains 4 kg of PCM (SSPCM), while the third one contains 4 kg of local clay (SSLC), and are presented previously in Figs. 6 and 7. The results provide water productivity, the solar radiation intensity, wind speed, humidity, ambient air temperature, glass cover temperature, basin water temperature, clay temperature, and phase change material temperature at every hour over a day period from seven o’clock in the morning to eight o’clock in the evening. Figure 8 shows the solar radiation intensity, ambient temperature, wind speed, and humidity with time. As seen in the figure, the solar radiation is gradually increased during the daytime from the morning, and then it is gradually decreased for the rest of the day. The maximum solar radiation is about 1099 W/m^2^ at 3 p.m. Moreover, it can be observed that the temperature of the ambient air is gradually increased during the daytime from the morning, and then it is gradually decreased for the rest of the day. The maximum ambient air temperature, wind speed, and humidity reached about 38 °C, 24 km/h, and 45%, respectively.

**Fig. 9.**
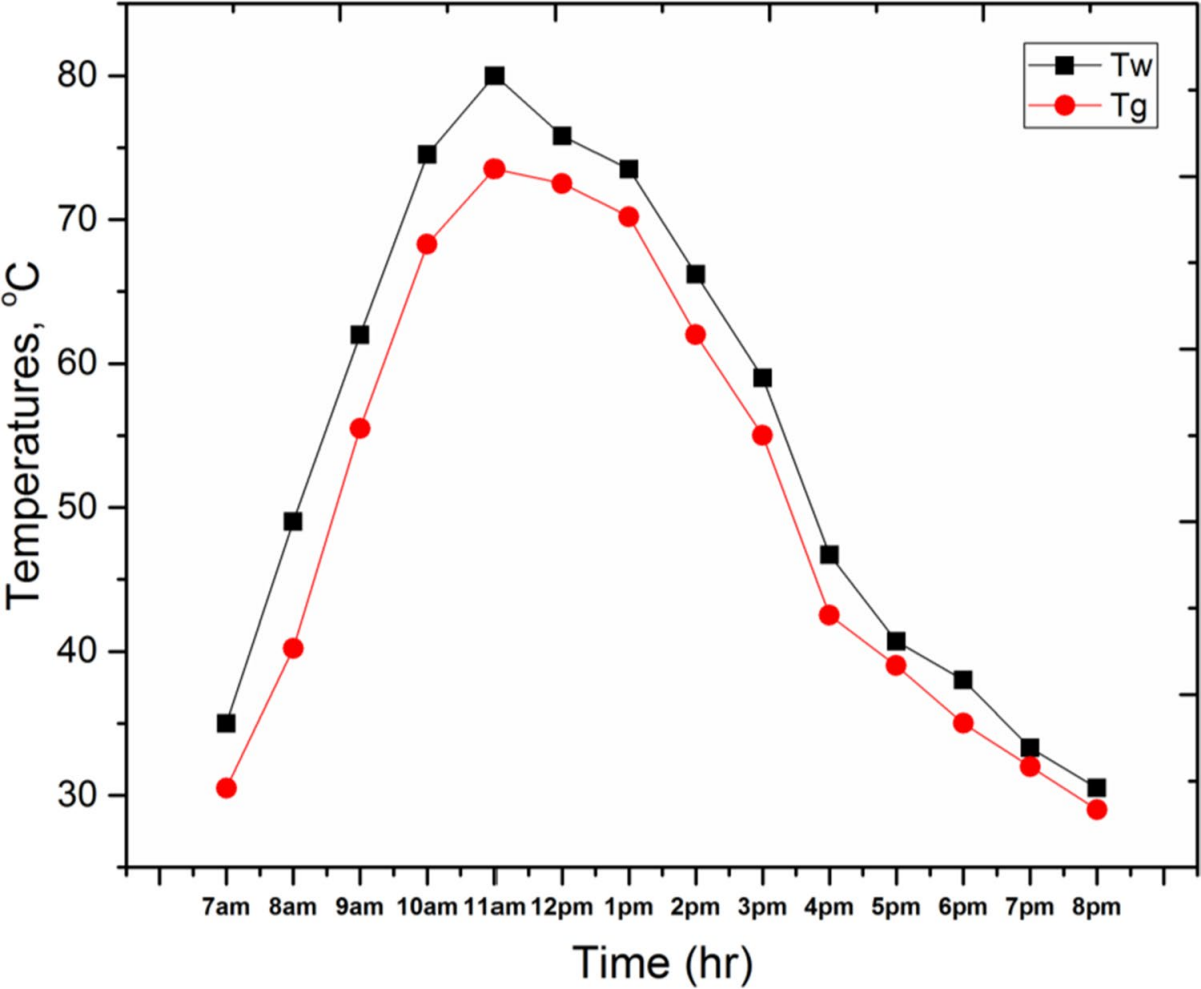
Basin water temperature (T_w_), and glass temperature (T_g_) for CSS

**Fig. 10.**
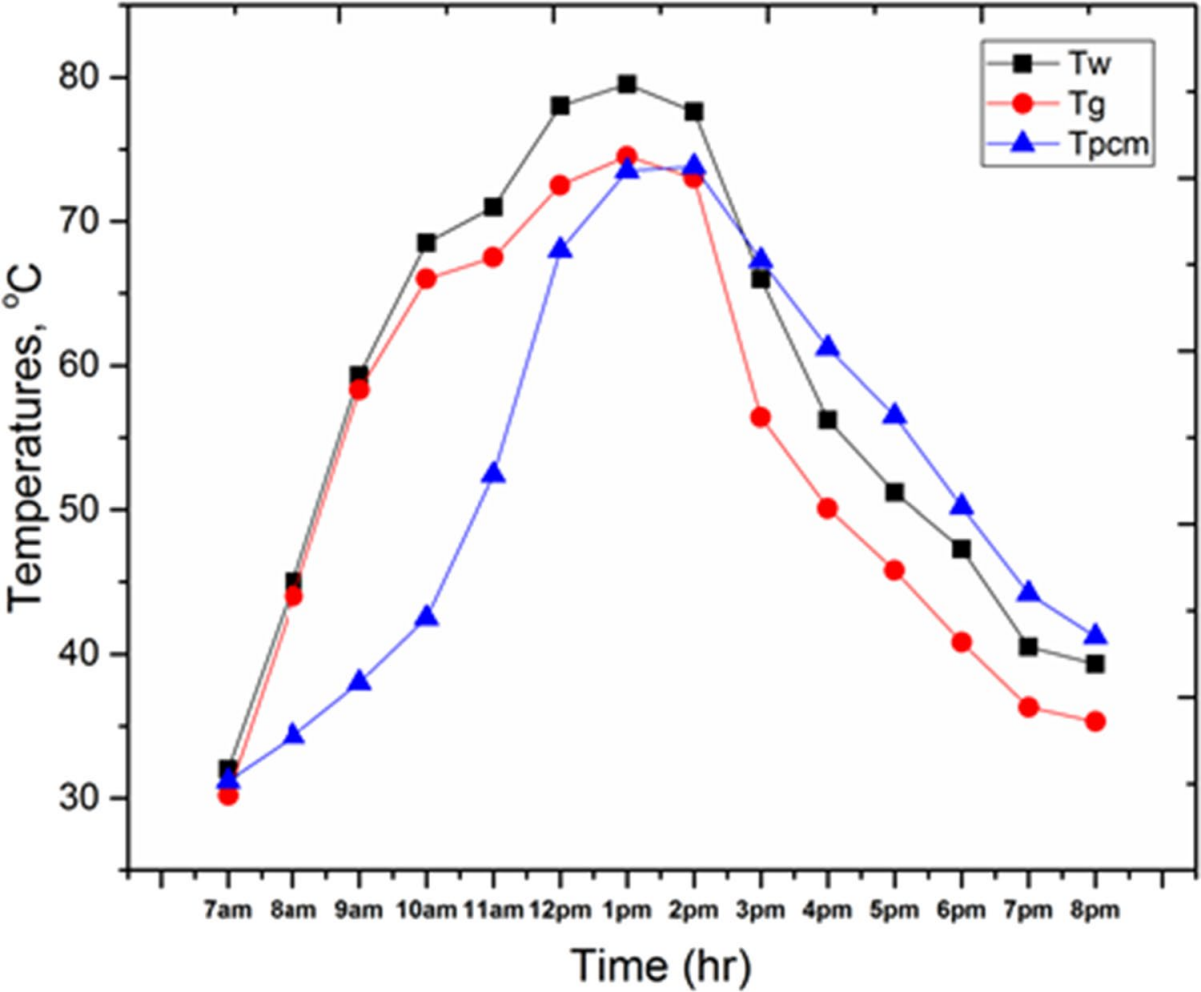
Basin water temperature (T_w_), glass temperature (T_g_), and PCM temperature (T_pcm_) for SSPCM

Figure 9 shows the variation of the following parameters through the test day, the basin water temperature (T_w_), and glass temperature (T_g_) for the conventional solar still (CSS). It can be seen from the figure that all temperatures are increased with time, starting from the early hours in the morning up to the maximum values at the time between 11 a.m. and 1 p.m. and then temperatures go down by the rest of the day. The maximum basin water temperature and glass temperature are 80 °C and 73.5 °C, respectively. Moreover, Fig. 10 shows the variation of the following parameters through the test day, the basin water temperature (T_w_), glass temperature (T_g_), and PCM temperature (T_pcm_) for the solar still with 4 kg of PCM inside basin area (SSPCM). It can be observed that all temperatures are increased with time, starting from the early hours in the morning up to the maximum value at the time between 12 p.m. and 2 p.m., then the temperatures go down by the rest of the day. The maximum basin water temperature, glass temperature, and PCM temperature are 79.5 °C, 74.5 °C, and 73.8 °C, respectively. Moreover, it can be detected that, through the period from 7 a.m. to 1 p.m., T_pcm_ has lower values than that for T_w_ and T_g_, while during the period of 3 p.m. to 8 p.m., T_pcm_ gives higher values than that for T_w_ and T_gn_ because of the energy stored inside the PCM was released.

**Fig. 11.**
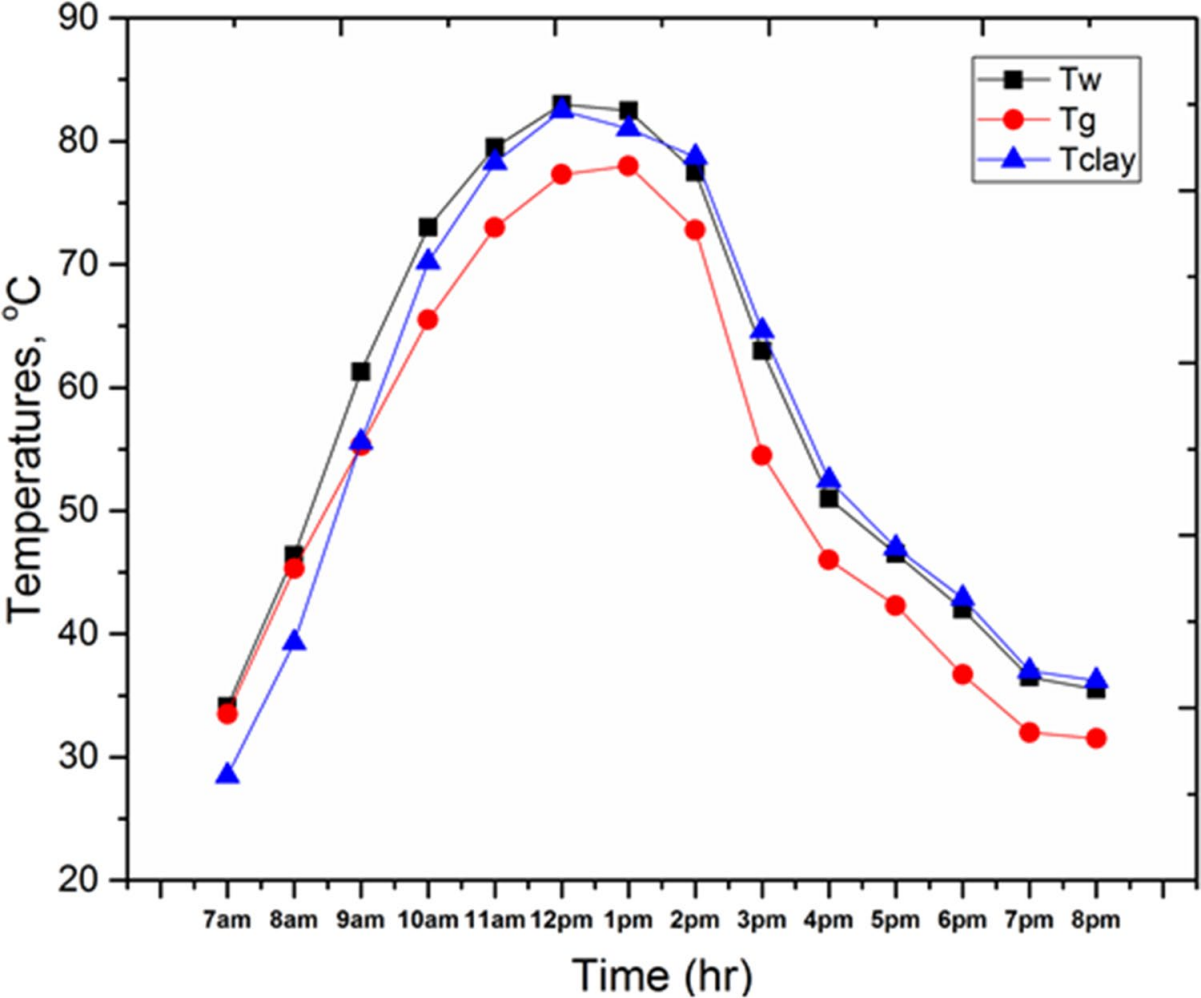
Basin water temperature (T_w_), glass temperature (T_g_), and clay temperature (T_clay_) for SSLC

Figure 11 shows the variation of the following parameters through the test day, the basin water temperature (T_w_), glass temperature (T_g_), and clay temperature (T_clay_) for the solar still with 4 kg of local clay material inside the basin area (SSLC). It can be observed that all temperatures are increased with time, starting from the early hours in the morning up to the maximum value at the time between 11 a.m. and 2 p.m., then the proposed temperatures are decreased during the rest period of the day. The maximum basin water temperature, glass temperature, and clay temperature are 83 °C, 78 °C, and 82.5 °C, respectively. Moreover, it can be seen from the figure that, through the period from 2 p.m. to 8 p.m., T_clay_ gives higher values than that for T_w_ and T_g_ because of releasing the energy stored inside the clay material.

**Fig. 12:**
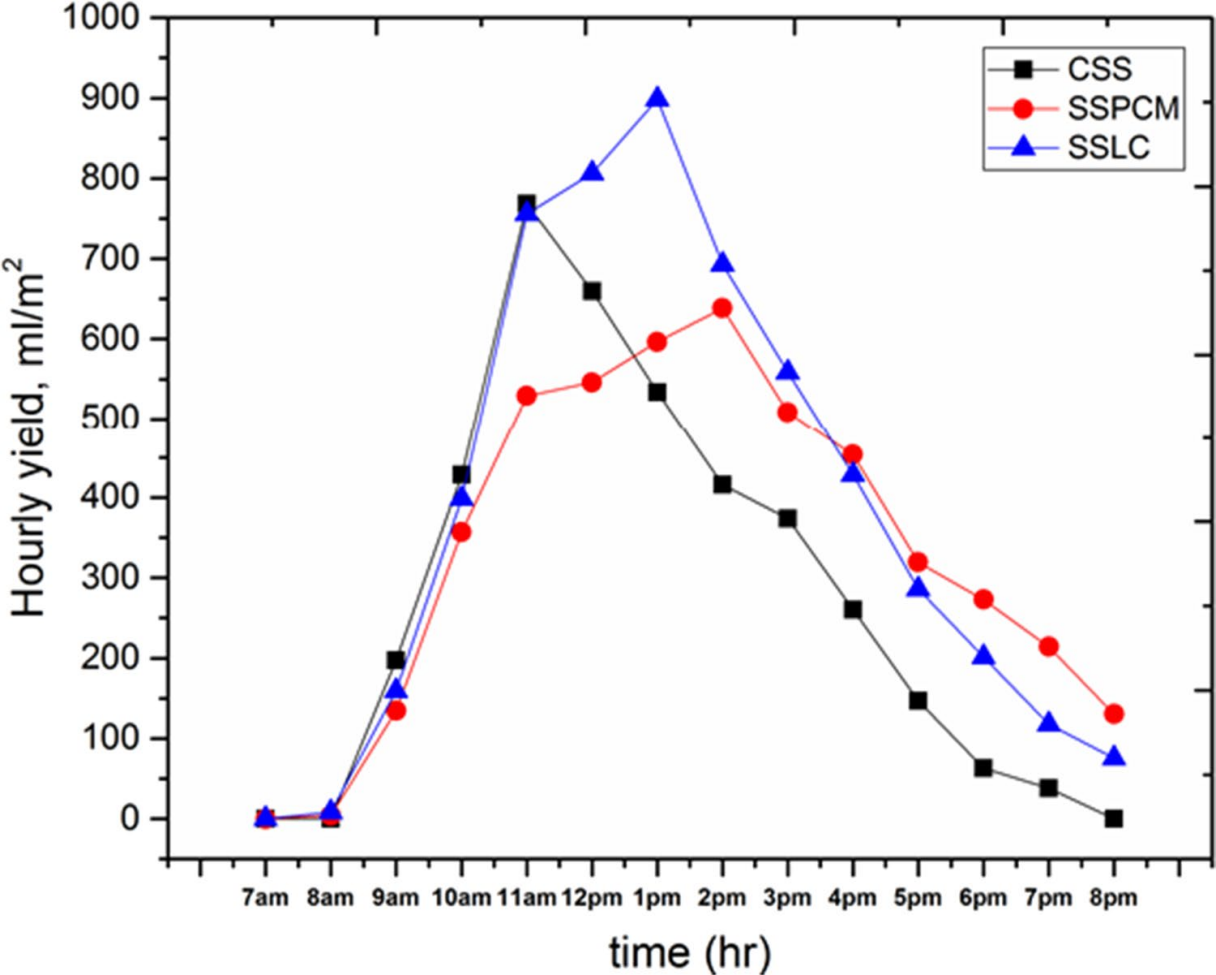
The hourly productivity for the CSS, SSPCM, and SSLC with time

**Fig. 13:**
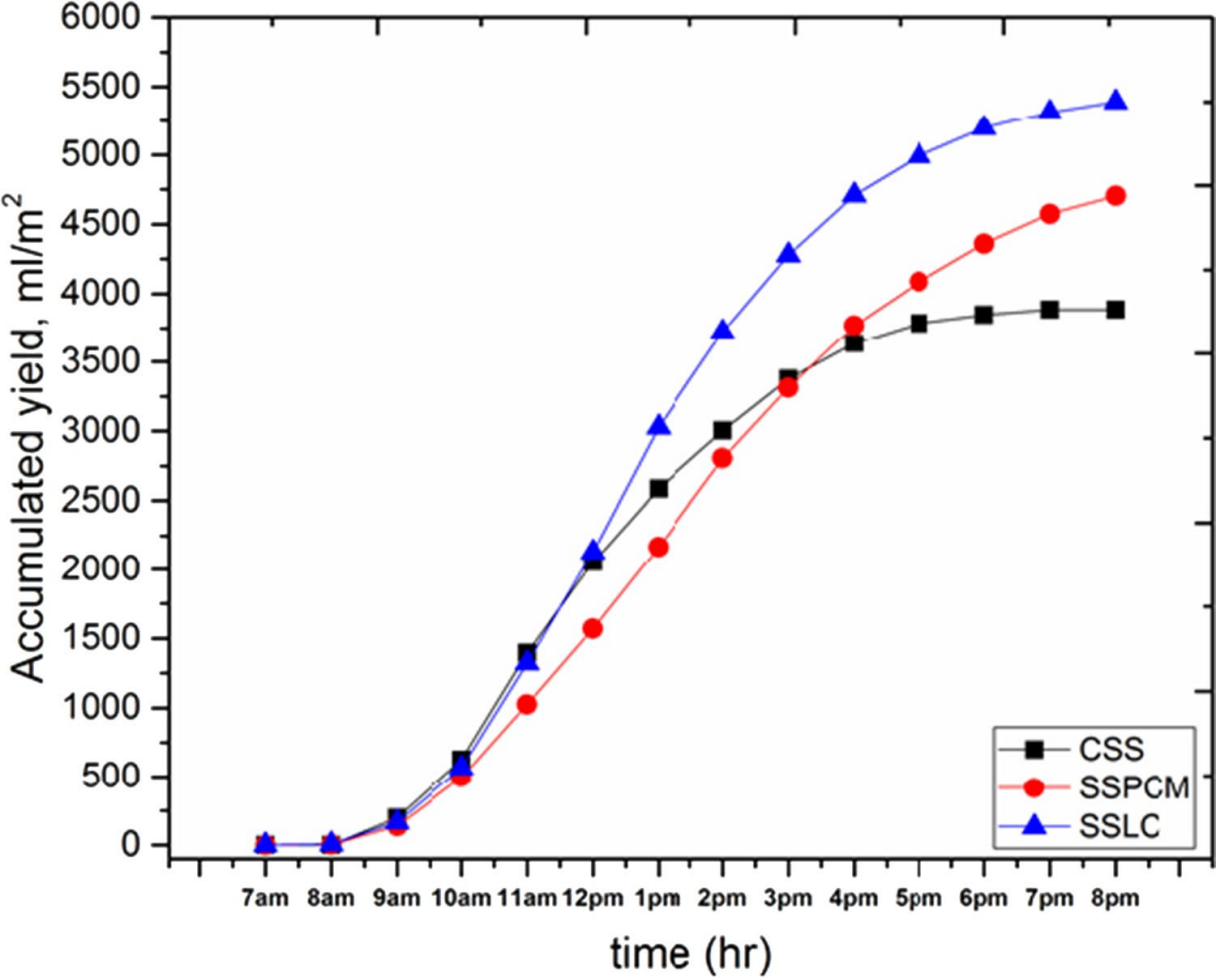
The accumulated freshwater productivity for the CSS, SSPCM, and SSLC with time

Figure 12 shows the hourly productivity per unit area of the three proposed cases for the conventional solar still (CSS), solar still with 4 kg PCM (SSPCM), and solar still with 4 kg clay (SSLC). It was observed that at 7 a.m. to 12 p.m., the hourly yield in the case of CSS and SSLC is slightly equal. However, the hourly productivity in the case of SSPCM was lower than that for the other solar stills due to the beginning of heat storing process in the used PCM. Moreover, through the interval time from 12:00 p.m. to the end of the test day, the hourly productivity of the CSS was lower than the other stills. Furthermore, the hourly productivity of the SSLC through the same interval was higher than the productivity of both the CSS and SSPCM, due to the efficient heat absorption by the used local clay, which led to the increase in evaporation rate. Figure 13 shows the accumulated freshwater productivity variation per unit area of the CSS, SSPCM, and SSLC. It can be observed that the total accumulated productivity of the CSS, SSPCM, and SSLC reached about 3885, 4704, and 5388.6 ml/m^2^, respectively. Compared to the CSS, the total production of drinking water was increased by about 21% when using the PCM, and 38.7% when using the local clay material. Furthermore, during the daytime, from 08:00 a.m. to 03:00 p.m., the productivity in the case of using SSLC was higher than the case of using CSS and SSPCM, respectively, and the productivity of the CSS was also higher than the productivity of SSPCM, because PCM began to store heat in the mentioned interval. From 3:00 p.m. to the end of the test day, the productivity of SSLC was continually increased with time. Moreover, the total productivity of the SSPCM is increased over the CSS because the stored energy inside PCM was released through this interval time.

**Fig. 14:**
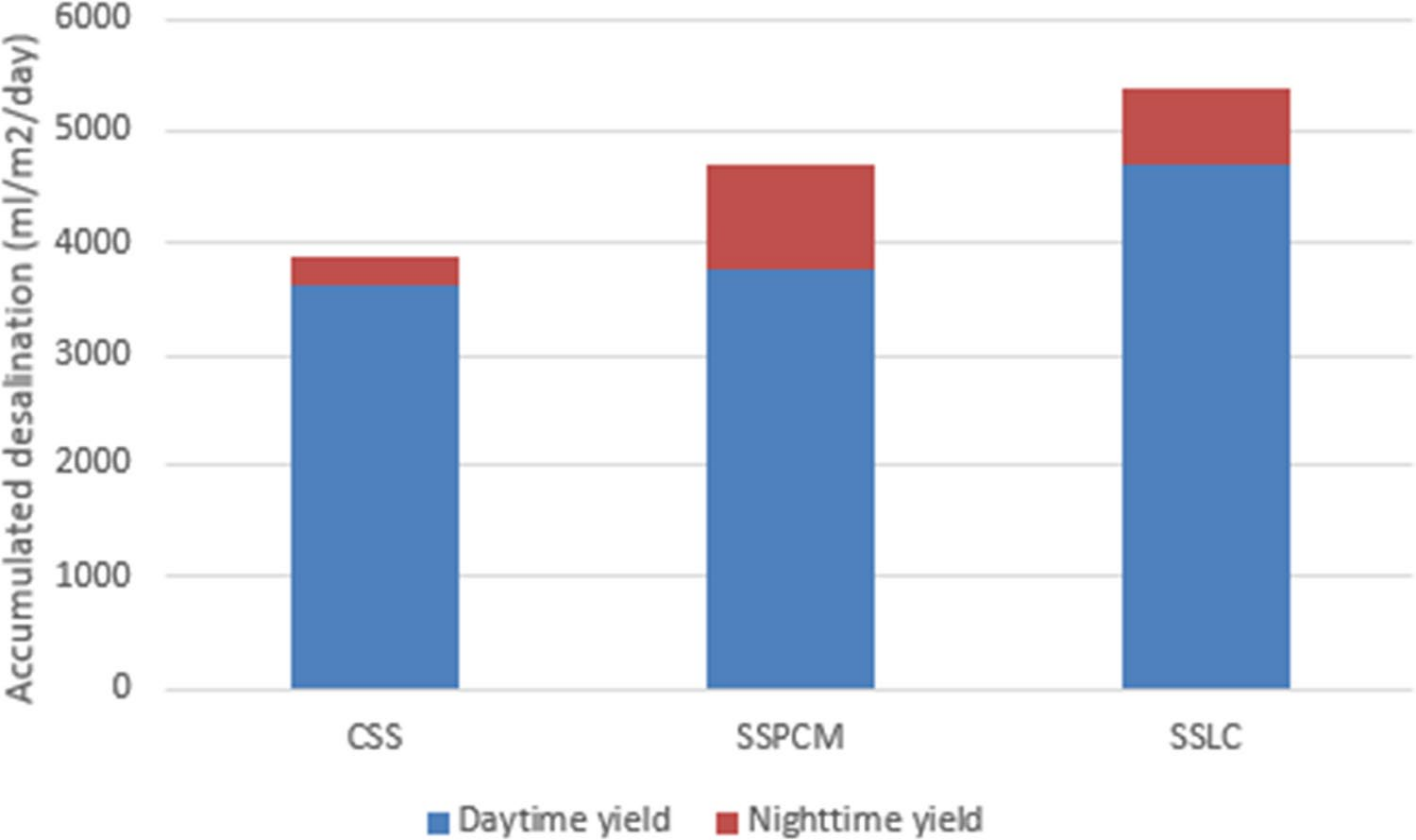
Daytime and nighttime accumulated productivity of the proposed solar stills

Figure 14 presents the proposed solar stills’ daytime and nighttime accumulated productivities. The daytime and nighttime productivities of the conventional solar still recorded lower values than that for the other stills, which reached at daytime 3637.2 ml/m^2^ and at nighttime 247.8 ml/m^2^. However, for the SSPCM, the nighttime productivity was higher than that for the other stills, which reached up to 936.6 ml/m^2^ at nighttime and the daytime productivity reached up to 3767.4 ml/m^2^. However, SSLC has recorded the highest daytime productivity, which reached about 4708.2 ml/m^2^ and the nighttime productivity reached up to 680.4 ml/m^2^.

### Solar still average daily efficiency and evaporative heat transfer coefficient (EHTC)

The average daily efficiency η_d_ for the proposed CSS, SSPCM, and SSLC was calculated and illustrated by using Eq. 4 (Shmroukh and Ookawara 2020) by multiplying the total hourly productivity per unit area m_p_ (kg/m^2^.day) and the latent heat of vaporization h_fg_ (J/kg) corresponding to the average temperature of the basin water T_w_ (°C) and divided by the daily average solar radiation intensity per unit area I(t) (W/m^2^) through the test period (7:00 a.m.to 8:00 p.m.).

4 $${\upeta}_d=\frac{\sum_{i=1}^{i=t}\left({\mathrm{m}}_{\mathrm{p}}\times {\mathrm{h}}_{\mathrm{fg}}\right)}{\mathrm{A}\times \sum_{i=1}^{i=t}\mathrm{I}\left(\mathrm{t}\right)\times 3600}$$

The latent heat of the basin seawater was calculated by Kabeel and Abdelgaied (2017) as follows:

5 $${\mathrm{h}}_{\mathrm{fg}}={10}^3\left(2501.9-2.40706\times Tw+1.192217\times {10}^{-3}\ Tw2-1.5863\times {10}^{-5}\ {Tw}^{{}^3}\right)$$

The basin seawater temperatures are recorded from the experiment and substituted in Eq. 5 to calculate the latent heat, then by using the total productivity, and the daily average solar radiation the efficiency can be calculated from Eq. 4.

**Fig. 15.**
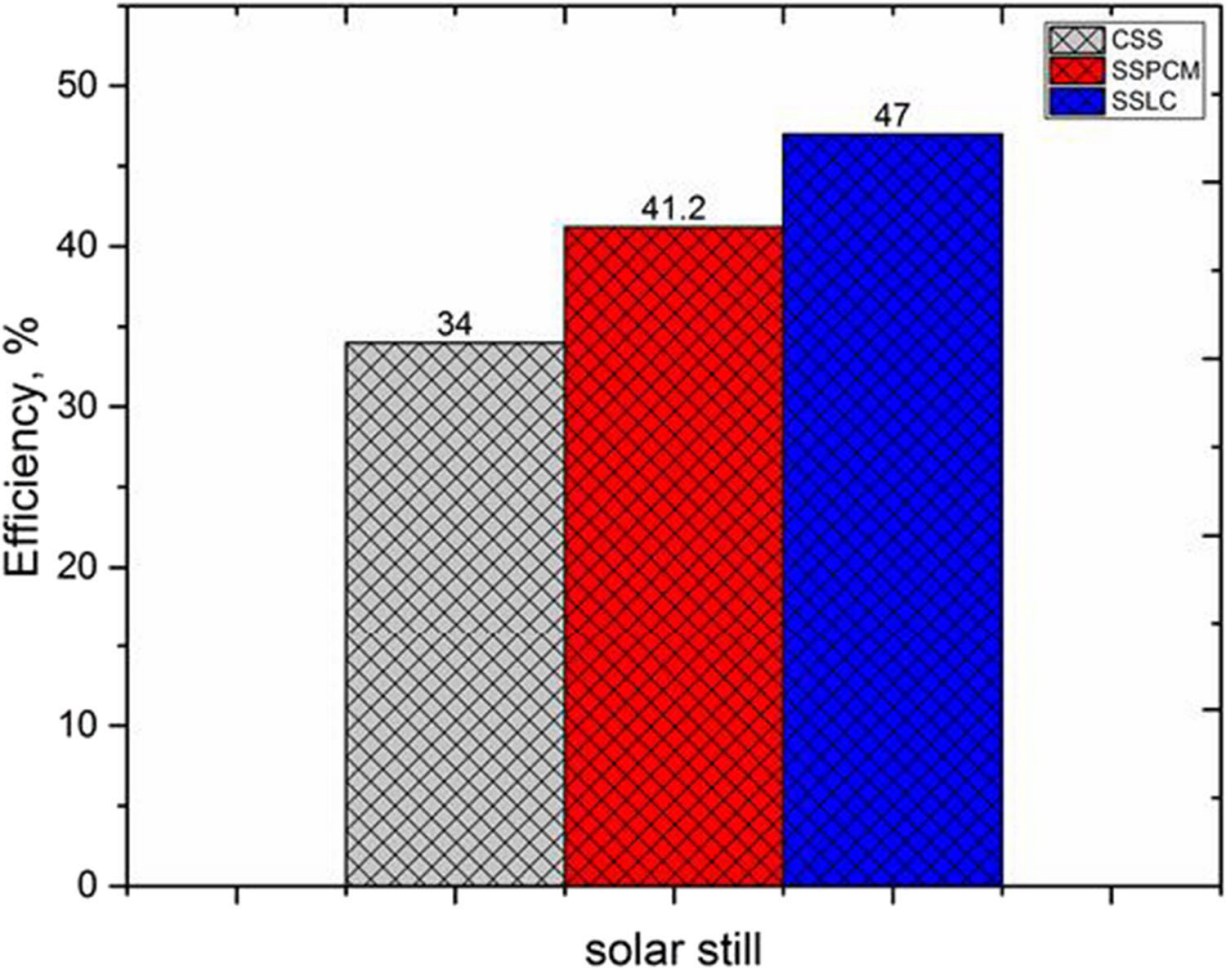
Average daily efficiency for the proposed solar stills

Figure 15 presents the average daily efficiency of the proposed solar stills. It is observed that the efficiency of SSLC was higher than CSS and SSPCM which reached up to 47%, 34%, and 41.2%, respectively.

### Economic analysis

The total fabrication cost, feed water cost, maintenance cost, and operating cost are affecting the payback period of the solar still (Jani and Modi 2019). Table 3 represents the cost per unit area of the proposed solar stills. As presented in Table 3, the total capital cost reach 41.9, 234.9, and 55.2 $/m^2^ for CSS, SSPCM, and SSLC, respectively, while the total productivity for CSS, SSPCM, and SSLC reached 3.885, 4.704, and 5.3886 l/m^2^ day, respectively. On the other hand, the average interest rate of lending banks in Egypt equals 12%, and the market price of water in Egypt is about 0.093 $/l, with 10 years as a life time of solar still (Shmroukh and Ookawara 2020; Mohammed et al. 2021). The salvage value of the system usually was taken 20% of the cost of usable material (Kumar and Tiwari 2009; Kabeel et al. 2010; Rahbar and Esfahani 2012), and the cost of feed water, operating cost, and maintenance cost is negligible (Jani and Modi 2019). Therefore, the total investment for CSS equals to 33.52 $/m^2^, for SSPCM equals 187.92 $/m^2^, and for SSLC equals 44.16 $/m^2^.

- The total gain for the day was about 0.093×3.885 l = 0.3613 $/m^2^ day for CSS, 0.44 $/m^2^ day for SSPCM, and 0.501 $/m^2^ day for SSLC.
- The payback period = investment/saving, it reaches about 93 days for CSS, 427 for SSPCM, and 88 days for SSLC.

**Table 3:** The cost per unit area of the solar stills

No.	Description	CSS $/m^2^	SSPCM $/m^2^	SSLC $/m^2^
1	Cost of the fabrication galvanized iron	31.7	31.7	31.7
2	Storage material (PCM or Clay)	---	193	13.3
3	Cost of glass	3.8	3.8	3.8
4	Cost of insulation	3.2	3.2	3.2
5	Black paint	1.9	1.9	1.9
6	Silicon seal agent	1.3	1.3	1.3
7	Total cost	41.9 $/m^2^	234.9 $/m^2^	55.2 $/m^2^

- The total production of solar still along its life = daily production × yearly operation period × lifetime = 3.885×340×10 = 13209 l/m^2^ for CSS.
- Consequently, the 1 l water production cost for the proposed solar stills is calculated as follows:
- Production cost = Total investment/ Total production, which reaches about 0.00254 $/L for CSS, 0.0117 $/L for SSPCM, and 0.00241 $/L for SSLC.

### Fresh and seawater properties

The desalinated water can be used in different daily life applications, due to its acceptable levels of water clarity, PH, total dissolved solids (TDS), and hardness (Organization 2006). As shown in Table 4, these properties were tested and approved by Qena Company of Water and Wastewater, Quality Sector, General Laboratories Department, Qena Central Laboratory.

**Table 4:** Desalinate and salty feed water properties

Property	Feed water	Desalinated water	Max accepted limits (Organization 2006)
pH	7.94	7.28	6.5-8.5
Hardness in mg/l	8960	74.2	200
Water clarity (NTU)	-	0.01	1
Total dissolved solids (TDS)	36538.46 ppm	122.6	500 ppm

Furthermore, Table 5 represents the comparison between the present experimental study and other related studies from the open literature. It can be observed that the tested solar stills of the present study give higher daily productivities than the compared studies from literature.

**Table 5:** Comparison of the present experimental work and other previous studies

Reference	Case	Daily production	Daily production rise
(Samuel et al. 2016)	CSS with spherical ball salt storage CSS with sponge CSS without storage material	3.7 kg/m^2^ 2.7 kg/m^2^ 2.2 kg/m^2^	68% 22.7% ----
(Vigneswaran et al. 2019)	CSS with two types of PCM CSS with one type of PCM CSS without storage material	4.4 kg/m^2^ 4.020 kg/m^2^ 3.680 kg/m^2^	19.6% 9.2% ----
(El-Sebaii et al. 2009b)	CSS with 10 kg of sand CSS without storage material	4.005 kg/m^2^ 2.852 kg/m^2^	40.4 % ----
Present experimental work	SSLC SSPCM CSS	5.3886 kg/m^2^ 4.704 kg/m^2^ 3.885 kg/m^2^	38.7% 21 % ----

## Conclusions

The experiments are conducted on single-basin single-slope solar still with PCM and Egyptian red clay material in the basin, compared with conventional solar still and tested under the climatic conditions of Qena, Egypt.

The following summaries can be drawn from this research:

- The results showed that the total drinking water production from the CSS, SSPCM, and SSLC are 3885, 4704, and 5388.6 ml/m^2^, respectively. Compared to the CSS, the production of drinking water was increased by 21% when using the PCM, and 38.7% when using the local clay material.
- The maximum accumulated productivity extracted from the SSLC reached up to 5.3886 l/m^2^.
- The average daily efficiency of the CSS, SSPCM, and SSLC reached 34%, 41.2% and, 47%, respectively.
- The simple payback period for the CSS, SSPCM, and SSLC reached about 93, 427, and 88 days of operation, respectively.
- Moreover, the produced desalinated water can be used in several life applications, due to its convenient ranges of total dissolved solids (TDS), water clarity, hardness, and PH.

## List of symbols

A: Area (m^2^)
h_fg_: Latent heat (J/kg)
I(t): Solar radiation per unit area (W/m^2^)
I: Number of measured values (–)
m_p_: Total yield per unit area (kg/m^2^ day)
N: Set of measured values (–)
R: Determined value (–)
T_g_: Glass temperature (°C)
T_pcm_: PCM temperature (°C)
T_W_: Water temperature (°C)
X: Value of the measured quantity (–)

## Greek symbol

𝛅: Uncertainty (–)
ƞ_d_: Average efficiency (–)
σ: Standard deviation (–)

## Abbreviations

CSS: Conventional solar still
PCM: Phase change materials
SSLC: Solar still with local clay
SSPCM: Solar still with phase change material
SSSS: Single slope solar still
TDS: Total dissolved solids
